# Comorbid obsessive-compulsive symptoms in schizophrenia: contributions of pharmacological and genetic factors

**DOI:** 10.3389/fphar.2013.00099

**Published:** 2013-08-09

**Authors:** Frederike Schirmbeck, Mathias Zink

**Affiliations:** Central Institute of Mental Health, Department of Psychiatry and Psychotherapy, Faculty Medicine Mannheim, Heidelberg UniversityMannheim, Germany

**Keywords:** antipsychotic agents, clozapine, comorbidity, compulsion, genetics, obsession, schizophrenia, SLC1A1

## Abstract

A large subgroup of around 25% of schizophrenia patients suffers from obsessive-compulsive symptoms (OCS) and about 12% fulfill the diagnostic criteria of an obsessive-compulsive disorder (OCD). The additional occurrence of OCS is associated with high subjective burden of disease, additional neurocognitive impairment, poorer social and vocational functioning, greater service utilization and high levels of anxiety and depression. Comorbid patients can be assigned to heterogeneous subgroups. One hypothesis assumes that second generation antipsychotics (SGAs), most importantly clozapine, might aggravate or even induce second-onset OCS. Several arguments support this assumption, most importantly the observed chronological order of first psychotic manifestation, start of treatment with clozapine and onset of OCS. In addition, correlations between OCS-severity and dose and serum levels and duration of clozapine treatment hint toward a dose-dependent side effect. It has been hypothesized that genetic risk-factors dispose patients with schizophrenia to develop OCS. One study in a South Korean sample reported associations with polymorphisms in the gene *SLC1A1* (solute carrier family 1A1) and SGA-induced OCS. However, this finding could not be replicated in European patients. Preliminary results also suggest an involvement of polymorphisms in the *BDNF* gene (brain-derived neurotrophic factor) and an interaction between markers of *SLC1A1* and the gene *DLGAP3* (disc large associated protein 3) as well as GRIN2B (N-methyl-D-aspartate receptor subunit 2B). Further research of well-defined samples, in particular studies investigating possible interactions of genetic risk-constellations and pharmacodynamic properties, are needed to clarify the assumed development of SGA-induced OCS. Results might improve pathogenic concepts and facilitate the definition of at risk populations, early detection and monitoring of OCS as well as multimodal therapeutic interventions.

## Gene and environment interactions in psychiatric disorders

Several frequent and disabling mental disorders manifest as a consequence of both genetic and environmental factors. Schizophrenia for instance is commonly perceived on the background of a gene-and-environment interaction (GxEI), where individual genetic properties dispose to a specific liability and sensitivity for specific stressors. These could include migration, other stressful life events, or effects of psychotropic substances (van Os and Kapur, 2009; van Os et al., 2008, 2010). Similar concepts were suggested regarding depression (Keers and Uher, 2012), anxiety disorders (Gregory et al., 2008; Nugent et al., 2011) and obsessive-compulsive disorder (OCD) (Nicolini et al., 2009; Pauls, 2010). Expanding the view to common comorbidities it is even more complex and demanding to investigate whether these might also be described on the basis of GxEI.

In this review we summarize evidence investigating possible pharmacological and genetic risk constellations underlying the co-occurrence of comorbid obsessive-compulsive symptoms (OCS) in schizophrenia.

## Epidemiology of OCS in schizophrenia

**Figure 1:**
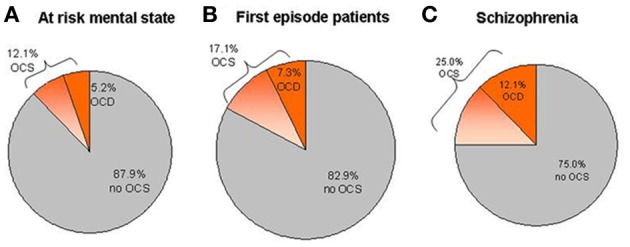
**Prevalence estimations of OCS and OCD within different samples of patients**. **(A)** Mean prevalence rates in ARMS studies. **(B)** Mean prevalence rates in first-episode psychotic patients. **(C)** Mean prevalence rates in patients suffering from chronic schizophrenia.

Patients with schizophrenia have a high lifetime risk of about 25% for comorbid OCS and a recent meta-analysis reports that 12.1% also fulfill the criteria for an OCD (Figure 1C; Poyurovsky et al., 2004, 2012; Buckley et al., 2009; Lysaker and Whitney, 2009; Mukhopadhaya et al., 2009; Achim et al., 2011; Hadi et al., 2011). In contrast, prevalence rates of 1–2% for OCD in the general population are considerably lower (Murphy et al., 2010). Accordingly, primary OCD-patients carry a relatively low risk (1.7%) to develop comorbid psychotic symptoms (de Haan et al., 2009).

Schizophrenia patients, who suffer from comorbid OCS often also display pronounced and sometimes treatment resistant positive and negative symptoms (Cunill et al., 2009; Sa et al., 2009). In addition, they present with specific neurocognitive deficits (Schirmbeck et al., 2012b), more often utilize health care services (Berman et al., 1995a), and show heightened levels of anxiety and depression (Lysaker and Whitney, 2009) when compared to schizophrenia patients without OCS. These pronounced impairments result in an additional burden of disease, in poorer social and vocational function (Fenton and McGlashan, 1986; Lysaker et al., 2004; Öngür and Goff, 2005; de Haan et al., 2013) and in a less favorable overall prognosis (Schirmbeck and Zink, 2013).

## Clinical presentation and explanatory concepts

Several heterogeneous subgroups of comorbid patients have been suggested depending on the diverse clinical course and phenotypic presentation. In order to unravel the specific interplay of genetic, psychosocial and pharmacological factors current research tries to focus on homogeneous subgroups. Subdivisions into such subgroups can for example be achieved according to the time point of first manifestation of comorbid OCS and the clinical course.

### Onset of OCS

First onset of OCS has been described at different stages during the course of psychotic illness:

1. Before psychosis as independent, co-existing symptoms or diagnosed OCD.
2. Prior to psychotic manifestation as part of the at risk mental state (ARMS).
3. Simultaneously with the first manifestation of psychosis.
4. After the first psychotic episode during the course of chronic schizophrenia.
5. As *de novo* OCS after initiation of antipsychotic treatment.

A remarkably large subgroup of patients already suffers from OCS during ARMS. Overall, sample-size weighted mean prevalence rates show that 12.1% (CI: 9.4–14.8%) of ARMS patients report OCS (Shioiri et al., 2007; Niendam et al., 2009; Bechdolf et al., 2011; Sterk et al., 2011; Hur et al., 2012), while 5.2% (CI: 4.1–6.3%) fulfill the criteria for OCD (Shioiri et al., 2007; Niendam et al., 2009; Rubino et al., 2009; Bechdolf et al., 2011; Fontenelle et al., 2011; DeVylder et al., 2012; Fusar-Poli et al., 2012; Sterk et al., 2011) (Figure 1A). Slightly higher averaged rates for OCS (17.1%, CI: 14.0–20.2) and OCD (7.3%, CI: 5.3–9.3%) can be found in first episode patients (Figure 1B; Poyurovsky et al., 1999a; de Haan et al., 2004; Sterk et al., 2011; de Haan et al., 2012; Zink et al., under review). Large variability of epidemiological data between studies can be explained by differences in the definition of ARMS criteria and differences in the psychometric assessment of OCS or OCD. Regarding the impact of OCS during the ARMS on other clinical variables, findings have been heterogeneous. Whereas higher impairment of psychosocial functioning (de Haan et al., 2012; DeVylder et al., 2012; Fusar-Poli et al., 2012; Hur et al., 2012) and more severe depressive symptoms (Niendam et al., 2009; DeVylder et al., 2012; Fontenelle et al., 2012; de Haan et al., 2013) have consistently been reported, findings regarding transition rates into psychosis (Niendam et al., 2009; Fontenelle et al., 2011, 2012; Fusar-Poli et al., 2012) and cognition (Van Dael et al., 2011){4854}(Hur et al., 2012) are contradicting.

**Figure 2:**
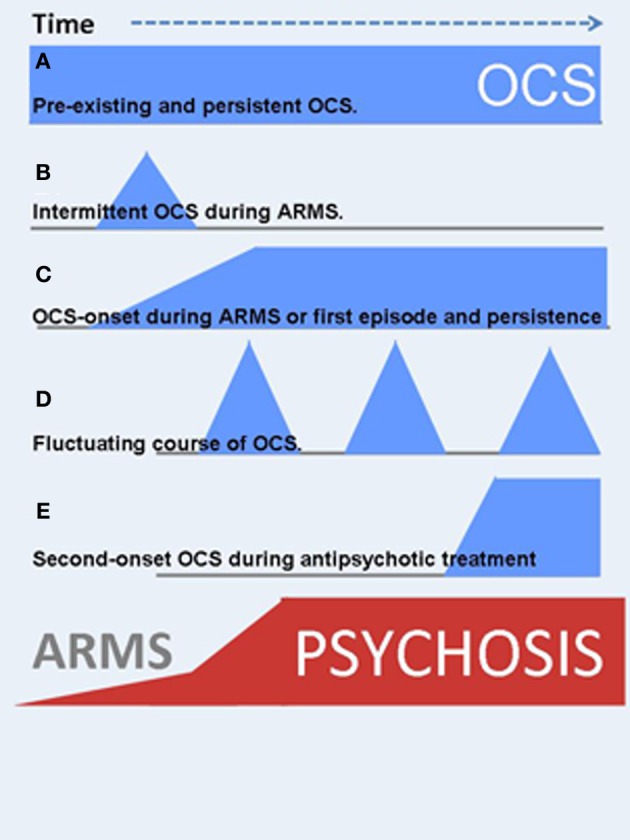
**OCS might manifest at different time points during the course of schizophrenia illness**. In addition, the clinical course is highly variable. Blue symbols indicate the onset and severity of OCS, red ones are related to psychotic symptoms. **(A)** Pre-existing and persistent OCS. **(B)** Intermittent OCS during ARMS or later in the clinical course. **(C)** OCS-onset during ARMS and persistent course, strongly associated to the psychotic symptoms (schizo-obsessive concept). **(D)** Fluctuating course of OCS. **(E)** Second-onset OCS during antipsychotic treatment.

Apart from OCS during the ARMS a growing body of evidence investigated the co-occurrence of OCS during manifest schizophrenia. A significant subgroup within these patients reports OCS development after treatment-start with second generation antipsychotic agents (SGA). The order of the three events “onset of psychosis,” “start with SGA treatment” and subsequent “*de novo* development of OCS” hints toward the involvement of pharmacodynamic mechanisms (see Figure 2E and detailed description in section OCS induced by second-generation antipsychotics).

### Clinical course

In addition to varying time-points of first manifestation of OCS, the course of symptom severity over time also differs (Figure 2). OCS may appear as fluctuating symptoms, they may resolve, persist or even worsen over time. Within those patients who already reported manifest OCD prior to the psychotic illness, e.g., as adolescents, OCS will most likely persist or worsen independent of the course of schizophrenia (Hwang et al., 2009). Within those individuals who develop OCS sometime during the course of schizophrenia, only scarce longitudinal studies examined quantitative changes over time. One large investigation from the Netherlands followed participants over a period of 5 years and described a predominantly fluctuating course of OCS severity in over 70% of the comorbid sample: Some patients experienced the remission of OCS, others a fluctuating, more or less cyclic course, some reported first onset of OCS, whereas a forth group showed persisting symptom severity (Mahasuar et al., 2011). Another longitudinal study in a German sample investigated two pharmacological diverse groups and found persisting OCS severity over 12 months in the group treated with clozapine (CLZ) and olanzapine (OLZ) (Schirmbeck et al., 2012b, 2013). The diverse clinical course adds to the heterogeneous clinical presentation and suggests an involvement of different environmental factors and/or symptom interactions in the longitudinal development of comorbid OCS in schizophrenia (see section Underlying neurobiological mechanisms and environmental factors).

Furthermore, specific symptom dimensions of schizophrenia might overlap with the obsessive-compulsive phenotype (Fink and Taylor, 2001), making a careful differentiation and classification of presented symptoms necessary. Especially in cases of catatonic schizophrenia (Fink, 2013), a reliable assessment with established psychometric scales such as the catatonia rating scale (CRS) (Bräunig et al., 2000) and the Yale-Brown-Obsessive-Compulsive Scale (YBOCS) (Woody et al., 1995; de Haan et al., 2006) is often difficult. Historically, a more precise characterization was achieved by an undisguised view on the natural long-term course of schizophrenia, for instance published by Karl Leonhard (Beckmann et al., 2000). These descriptions allow clear discrimination between OCS and catatonic symptoms most importantly in patients with so-called “manneristic catatonia.”

Clinical research and an improved pathogenic understanding of OCS in patients with schizophrenia is thus dependent on a careful exploration of symptoms. Several aspects help to discriminate delusions or hallucinations from typical OCS to ensure valid and reliable diagnosis (see Table 1).

**Table 1:** **Identification of obsessive-compulsive symptoms in schizophrenia**.

Insight-criterion	Patients suffering from OCD typically fulfill three symptom characteristics: they attribute the obsessions, impulsive symptoms and compulsions to their own thinking, declare with insight their unreasonableness and show some degree of resistance against them. In particular the first two properties allow a differentiation from hallucinations and delusions. Ruminations or stereotypic ego-dystonic cognitions with direct relation to the contents to psychotic thinking should not be diagnosed as obsessions.
OCS not solely related to the psychotic content	Cleaning or checking behavior should be diagnosed as compulsions only if it is accompanied by typical obsessions and not, if the patient currently suffers from delusions of contamination, intoxication or infection.
Re-evaluation of OCS after remission of psychotic symptoms	If first manifestation of OCS occurs simultaneously with the first psychotic exacerbation, the final decision on a valid comorbid condition should be postponed until the remission of psychotic symptoms.
Differentiation from catatonic symptoms	Repetitive behavior or stereotypic actions should carefully be discriminated from catatonic symptoms most importantly in patients with so-called “manieristic catatonia.”
Obsessions presented as pseudohallucinations	A subgroups of OCS patients, who experience their obsessions as extremely aversive and burdening may try to distance themselves by using expressions such as “voices” or “foreign thought content”, but in most cases these phenomena can be characterized as pseudohallucinations.
SGA-induced OCS	Patients without a previous history of OCS might develop these phenomena during antipsychotic treatment. This constellation hints toward the unfavorable effect of second-onset OCS induced by SGAs.

*Clinical aspects that have to be considered when differentiating between psychotic symptoms (delusions, hallucinations) and OCS*.

### Pathogenic concepts

In attempts to explain the co-occurrence of OCS in schizophrenia, heterogeneous, partly overlapping but also contradicting pathogenic concepts have been suggested.

The two rather common psychiatric syndromes could of course develop independently, representing a random association. Based on the above mentioned high prevalence rates and diverse clinical presentation this cannot be the only explanation for OCS in nearly every fourth patient with schizophrenia.

Early concepts assumed that patients with schizophrenia develop OCS as an attempt to reduce psychotic symptoms and thus, the presence of OCS was proposed to have protective effects regarding psychotic disintegration, based on single-case analyses or small case series (Stengel, 1945; Dowling et al., 1995). Similarly, Guillem et al. described negative correlations between specific OCS and the severity of psychotic disorganization in thinking and behavior, proposing compensating mechanisms (Guillem et al., 2009). However, in a broader perspective, subsequent research revealed higher severity of psychotic symptoms and more functional impairment if OCS were present (Cunill et al., 2009) (see above).

Approaching the co-occurrence from the OCD spectrum, the concept of “schizotypic OCD” has been described (Poyurovsky and Koran, 2005; Poyurovsky et al., 2008). This concept assumes that primary OCD-patients present beliefs, which can be classified on a spectrum between obsessions and delusions emphasizing the similarities as being irrational thoughts, the first with insight and the latter lacking insight. In line with this concept, the category of “obsessions without insight” was integrated into the fourth edition of the Diagnostic and Statistical Manual (DSM IV). OCD-patients without insight might therefore represent a subgroup with genetic, phenotypic and therapeutic vicinity to the schizophrenia-like spectrum (Tumkaya et al., 2009; Catapano et al., 2010).

Approaching the co-occurrence from the schizophrenia spectrum, a so-called “schizo-obsessive” subtype of psychosis has been proposed, based on cross-sectional evaluations (Poyurovsky, 2013). This subtype has been suggested to comprise OCS in addition to positive, negative and cognitive schizophrenia symptoms (Poyurovsky et al., 2012). Similar concepts have been proposed by Hwang et al. (2000), Bottas et al. (2005), and Reznik et al. (2001, 2005). Attempts to validate the “schizo-obsessive” subtype on a neurobiological level have been inconsistent. Some studies proposed specific neurological features (Sevincok et al., 2006; Poyurovsky et al., 2007b), cognitive deficits (Lysaker et al., 2002, 2009) and even structural abnormalities (Gross-Isseroff et al., 2003).

The mentioned high prevalence rates of OCS in the ARMS, led to the perception that specific OCS could be a part of the basic symptom cluster in the early course of schizophrenia (Sullwold and Huber, 1986; Ebel et al., 1989).

The summarized pathogenic concepts mirror the high degree of heterogeneity within the comorbid sample. They are currently discussed and the number of publications on this topic nearly doubles every year.

### Underlying neurobiological mechanisms and environmental factors

#### Neurobiology

While the described explanatory concepts mainly follow a clinical or psychopathological rationale, several investigations tried to improve the pathogenic understanding from a neurobiological perspective. So far, most emphasis has been given to a multimodal neurocognitive characterization. Preliminary investigations of neurological soft signs (Sevincok et al., 2006; Poyurovsky et al., 2007b) and neuroimaging techniques (Gross-Isseroff et al., 2003) need replication.

For primary OCD recent reviews of published literature reported specific cognitive deficits especially in the areas of cognitive shifting abilities, inhibitory control and the application of effective planning strategies (Kuelz et al., 2004). Based on these findings, the question arose whether OCS in schizophrenia might also be linked to additional cognitive impairment in these OCD-related domains (Lysaker and Whitney, 2009). Subsequently, several authors tried to differentiate schizophrenia samples with vs. without comorbid OCS on the basis of their neuropsychological performance. Findings have been contradicting. Whereas some investigations did not find any significant differences (Hermesh et al., 2003; Whitney et al., 2004; Öngür and Goff, 2005; Tumkaya et al., 2009; Tiryaki and Ozkorumak, 2010; Achim et al., 2011; Meijer et al., 2013), others even suggested that OCS may be associated with better cognitive abilities (Lee et al., 2009; Borkowska et al., 2013), especially in the prodromal states of schizophrenia (Van Dael et al., 2011; Fontenelle et al., 2012; Hur et al., 2012; Zink et al., under review). Most results, however, showed more pronounced deficits in the described domains of executive functioning (Hwang et al., 2000; Lysaker et al., 2002, 2009), cognitive flexibility (Kumbhani et al., 2010; Patel et al., 2010), and also delayed visual memory (Berman et al., 1998; Schirmbeck et al., 2011).

In a recent longitudinal assessment, Lysaker et al. prospectively analyzed executive functioning and reported that deficits were linked to greater concurrent and prospective self-report of OCS among schizophrenia patients (Lysaker et al., 2009). A comprehensive prospective investigation by Schirmbeck et al. explicitly included OCD-related cognitive domains in their analysis (Kuelz et al., 2004; Rajender et al., 2011). Over a period of 12 months schizophrenia patients with comorbid OCS showed significant pronounced deficits with increasing effect sizes regarding cognitive flexibility, visuo-spatial perception, and visual memory. Performance in these domains correlated with OCS severity (Schirmbeck et al., 2012b).

Based on these findings and with respect to possible causal pathways, it has been proposed that pronounced cognitive deficits reflect an underlying neurobiological risk factor for schizophrenia patients to develop OCS and mirror at least partially overlapping neurobiological mechanisms with OCD. In order to further substantiate this hypothesis neurobiological links that explain the pronounced deficits in the comorbid sample should be identified. Therefore, research should focus on candidate regions, which have been described in primary OCD, such as increased activation-levels in the orbitofrontal cortex (Whiteside et al., 2004; Friedlander and Desrocher, 2006)using fMRI approaches.

Regarding neurotransmission, current pathogenic theories of OCD assume a central serotonergic dysfunction in a network comprising cortical, striatal and thalamic centers (Pogarell et al., 2003). Corresponding evidence is provided by the therapeutic effects of selective serotonin reuptake inhibitors (SSRIs) and cognitive behavioral therapy (CBT) in OCD (Linden, 2006; Saxena et al., 2009). In addition, neuroimaging studies with structural and functional methods confirmed alterations in the suggested network (Friedlander and Desrocher, 2006; Menzies et al., 2008; Kwon et al., 2009a). Based on these findings it has been assumed that the strong serotonergic antagonism of CLZ (Coward, 1992; Meltzer and Huang, 2008; Meltzer, 2012) and OLZ (Duggan et al., 2000) constitute a pathogenic mechanism in the development of second-onset OCS in schizophrenia (for more detail see section Epidemiological evidence and Pharmacological evidence). However, apart from serotonergic dysfunctions, alterations in dopaminergic activity (Van der Wee et al., 2004) and in glutamatergic neurotransmission, have also been related to OCD: Support for the involvement of glutamate in the development of OCD comes from animal models (Joel, 2006; Albelda et al., 2010; Yang and Lu, 2011), human MR spectroscopy (Whiteside et al., 2006; Starck et al., 2008), treatment approaches addressing the glutamatergic system (Coric et al., 2005; Poyurovsky et al., 2005, 2010; Lafleur et al., 2006; Pittenger et al., 2006) and the following results from genetic investigations.

#### Genetic disposition

Previous family and twin studies suggest a strong heritability of OCD (Nicolini et al., 2009; Pauls, 2010). In contrast, results from genetic association studies with a primary focus on candidate genes of serotonergic and dopaminergic neurotransmission were rather ambiguous. So far, only one linkage finding has consistently been replicated, which refers to single nucleotide polymorphisms (SNP) in the gene *SLC1A1* (solute carrier family) on chromosome 9p24, encoding the neuronal glutamate transporter EAAC1 (excitatory amino acid carrier 1) (Veenstra-VanderWeele et al., 2001; Arnold et al., 2006; Dickel et al., 2006; Stewart et al., 2007; Shugart et al., 2009; Wendland et al., 2009).

Possible neurogenetic disposition to develop OCS during the course of psychotic illness has just recently become a focus of interest. Research within this field is still scarce and needs further exploration. Progress has been achieved within a specific subgroup, suggesting a genetic disposition to develop OCS during SGA treatment (see section Genetic disposition).

#### Environmental factors

As briefly mentioned above, the majority of comorbid patients reports large fluctuation of OCS severity as either remitting, *de novo* development or intermittent OCS. However, the effect of environmental factors on onset or symptom severity as well as on interactions with other psychopathological processes has scarcely been investigated. Thus, the small number of longitudinal studies leaves important aspects unresolved. These include the following questions: (1) Do dynamic OCS and psychotic symptoms follow a parallel course? (2) Does a causal interaction of symptom variability exist? (3) Does experienced stress, life-events or antipsychotic medication influence the severity and course of OCS? Detailed follow-up analyses of the potential influence of environmental factors are therefore needed. Patients, who recently reported changes in their OCS should be investigated by means of an ‘Experience Sampling Method’ (ESM). This approach captures the reactivity to environmental factors and the course of symptoms in detail on a day to day basis, in real life situations, which allows to resolve symptom interactions and contextual triggers of variability. Results could provide the basis for individualized interventions, including adjusted modules of cognitive behavioral therapy (CBT).

Inconsistent results regarding associated neurobiological and environmental factors are most probably a consequence of the reported heterogeneity within the comorbid sample. Furthermore, methodological concerns such as the restriction to mainly cross-sectional evaluations and a lack of power due to small sample sizes add to inconclusive findings.

Thus, progress in pathogenic understanding seems most likely if future research focuses on the detailed characterization of homogeneous subsamples. One recent very promising approach has been achieved within the subgroup of patients who develop secondary OCS during SGA treatment. The following section summarizes evidence supporting the hypothesis of SGA-induced OCS and introduces possible genetic risk factors.

## OCS induced by second-generation antipsychotics

The subgroup of patients who report first onset or aggravation of OCS after psychotic manifestation and treatment initiation with SGAs has been briefly mentioned above. The simple assessment of the order of three important events (first psychotic manifestation, start of antipsychotic treatment and subsequent onset of OCS) helps to define this subgroup (Lykouras et al., 2003; Schirmbeck and Zink, 2012; Schirmbeck et al., 2013). The observation that schizophrenia patients develop OCS after psychotic manifestation and treatment initiation is mainly linked to SGAs and has rarely been reported under first generation antipsychotics. Several authors related this observation to the fact that SGAs carry the important pharmacodynamic feature of balanced antidopaminergic and antiserotonergic properties, which markedly exceed the low affinity of first generation antipsychotics to serotonergic receptors (Meltzer, 1995; Meltzer et al., 2003). In addition, differential effects on GABAergic and glutamatergic neurotransmission have to be considered (Lopez-Gil et al., 2010).

The hypothesis of SGA-induced OCS as a side-effect (Lykouras et al., 2003; Kwon et al., 2009b) first arose after the pioneer observations of Baker et al. (1992) and de Haan et al. (1999). Since then several studies show a clear association and possible causal interaction between SGA-treatment, most importantly CLZ (Schirmbeck and Zink, 2012), and the *de novo* occurrence of OCS (de Haan et al., 2004; Reznik et al., 2004; Kwon et al., 2009b; Schirmbeck et al., 2011).

Without a doubt, CLZ must be considered an indispensable part of the antipsychotic armament (Joober and Boksa, 2010; Kang and Simpson, 2010; Kane, 2011; Meltzer, 2012), especially in cases with otherwise treatment resistant psychoses (Kane et al., 1988). Several investigations (Asenjo Lobos et al., 2010) including the CATIE-study (McEvoy et al., 2006) have demonstrated its superior antipsychotic efficacy (Gupta and Daniel, 1995; Still et al., 1996; Kelly et al., 2003). Therefore, CLZ is considered the antipsychotic of first choice in treatment resistant schizophrenia. In addition, the substance demonstrates important anti-suicidal effects resulting in low mortality rates of CLZ-treated schizophrenia patients (Tiihonen et al., 2009). However, within a variety of other important side effects (Asenjo Lobos et al., 2010), the *de novo* occurrence or exacerbation of OCS under antipsychotic treatment has most often been observed with CLZ (Lykouras et al., 2003; Reznik et al., 2004; Schirmbeck and Zink, 2012). Due to a lack of controlled clinical trials, proposed causal interrelations cannot be confirmed, according to the general criteria suggested by Bradford Hill (2011). Nevertheless, several epidemiological (Epidemiological evidence) and pharmacological (Pharmacological evidence) arguments support this assumption (for summary see Table 2).

**Table 2:** **Arguments supporting SGA-induction of OCS**.

Epidemiology	The prevalence rates of OCS in schizophrenia increased after market approval of clozapine (Schirmbeck and Zink, 2012).
	The comorbidity rates in later disease stages are higher than at first manifestation of schizophrenia (see Figure 1).
	Schizophrenia patients with comorbid OCS are most frequently found to be treated with clozapine. Vice versa high OCS prevalence in patients treated with clozapine (Poyurovsky et al., 2004; Lim et al., 2007; Poyurovsky et al., 2007a; Schirmbeck et al., 2011).
Pharmacology	The type of antipsychotic treatment is associated with the risk for OCS: Marked difference between samples treated with first generation antipsychotics or mainly dopaminergic SGAs (such as aripiprazole or amisulpride) compared to clozapine (Ertugrul et al., 2005; Sa et al., 2009; Schirmbeck et al., 2011).
	OCS manifest as a unfavorable drug effect *de novo* during treatment with potent antiserotonergic SGAs such as clozapine (see Figure 3) (Schirmbeck and Zink, 2012).
	The severity of OCS is positively correlated with duration, dosage and serum levels of clozapine treatment (Lin et al., 2006; Schirmbeck et al., 2011).
	The OCS severity is found stable over time in patients under stable clozapine treatment (Schirmbeck et al., 2013).
	The severity of OCS improves after reduction of clozapine dosage to minimally sufficient levels (due to augmentation or combination) (Rocha and Hara, 2006; Zink et al., 2006; Englisch et al., 2009).

*Summary of epidemiological and pharmacological arguments supporting the assumption that OCS can be induced or at least markedly aggravated by SGA-treatment as an unfavorable side effect (Schirmbeck et al., 2012b)*.

### Epidemiological evidence

#### Increase of OCS prevalence after market approval of SGAs

Only few investigations reported comorbidity rates in samples treated with first generation antipsychotics (Fenton and McGlashan, 1986; Berman et al., 1995a,b; Nolfe et al., 2010). After market approval of SGAs, most importantly CLZ, in the 1970s in Europe and the late 1980s in the USA (Hippius, 1989; Kang and Simpson, 2010), prevalence estimations markedly rose. Although a potential publication bias and increased general awareness of this topic needs to be considered, these data provide a first indirect hint toward a possible interrelation.

#### Higher OCS prevalence during the chronic course of schizophrenia

As mentioned, prevalence estimations on OCS and OCD in ARMS and first episode samples clearly vary (see Figure 1 and section Onset of OCS). However, when compared to established rates of 12% (OCD) and 25% (OCS) in chronic schizophrenia, they appear to be significantly smaller. The higher rates in the later stages of the disease might partly be attributed to antipsychotic treatment.

#### Onset of de novo OCS during antipsychotic treatment or marked aggravation

**Figure 3:**
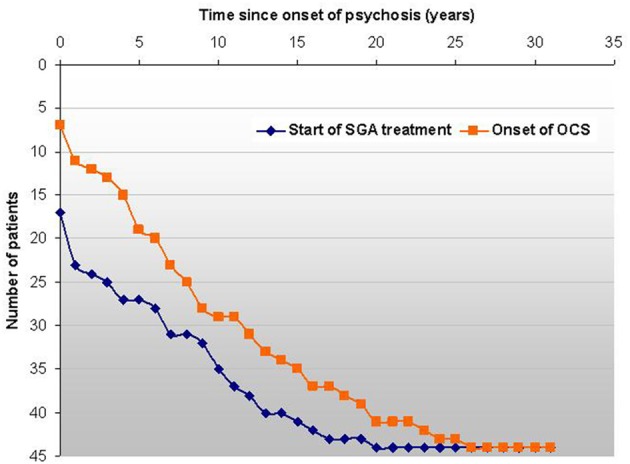
**In this sample of 44 comorbid patients the survival-curve shows the events “start of SGA treatment” and “first onset of OCS” on an individual basis related to the first manifestation of psychosis**. The longitudinal course of OCS shows a high degree of variability and is not depicted here. The graphs shows that in all but 7 cases OCS developed subsequently to psychosis and the vast majority of patients reported OCS onset after treatment-start with SGAs (Schirmbeck et al., 2012a).

Several case reports and cases series, as well as systematic evaluations describe the *de novo* emergence of OCS during the treatment with atypical antipsychotics, most importantly CLZ (Schirmbeck and Zink, 2012). Poyurovski et al. estimated that up to 70% of schizophrenia patients treated with mainly antiserotonergic SGAs such as CLZ, OLZ or risperidone develop secondary OCS (Poyurovsky et al., 2004), while Lykouras et al. reviewed published data and even reported *de novo* OCS in 77% of CLZ treated patients (Lykouras et al., 2003). Independent studies reported high proportions of SGA-induced OCS within samples of comorbid patients: 25 of 28 (89%) (Schirmbeck et al., 2011), 29 of 39 (74%) (Lin et al., 2006) and 23 of 26 (88%) (Lim et al., 2007). Furthermore, retrospective assessments of the individual disease histories show that most patients experience the onset of OCS after first manifestation of psychosis and the start with SGA-treatment (Schirmbeck et al., 2012a) (Figure 3).

### Pharmacological evidence

A variety of studies contribute to the assumption that antiserotonergic SGAs have pro-obsessive effects.

#### Higher prevalence of OCS in samples treated with CLZ

The risk for comorbid OCS markedly differs if patients are stratified according to their mode of antipsychotic treatment. As reported, high prevalence rates in CLZ-treated patients (Ertugrul et al., 2005), contrast with low rates during treatment with first generation antipsychotics, for instance, haloperidol (Sa et al., 2009) or other SGAs. Differences in pharmacodynamic properties, in particular regarding inherent serotonergic blockade, monoaminergic reuptake inhibition or even partial serotonergic agonism might explain these diverging findings (Shapiro et al., 2003; Meltzer and Huang, 2008; Meltzer and Sumiyoshi, 2008; Remington, 2008; Lopez-Gil et al., 2010). Interestingly, Aripiprazole (APZ), a partial dopaminergic and serotonergic agonist, was associated with an inherent anti-obsessive effect in schizophrenia patients with OCS (Connor et al., 2005; Zink et al., 2006; Chang et al., 2008; Englisch and Zink, 2008; Englisch et al., 2009), quite similar to amisulpride (AMS), a dopamine D3/D2 receptor antagonist (Kim et al., 2008; Pani et al., 2008). A comparison of schizophrenia patients under antipsychotic monotherapy with either mainly antiserotonergic SGAs (CLZ or OLZ; group I) or mainly dopaminergic SGAs (AMS or APZ; group II) revealed that more than 70% of group-I-patients suffered from OCS while less than 10% of patients in group-II reported OCS (Schirmbeck et al., 2011). *Vice versa*, a stratification of schizophrenia patients according to presence or absence of comorbid OCS revealed that 77% of comorbid patients were treated with clozapine while only 36% of schizophrenia patients without OCS received this substance (Lim et al., 2007). These results clearly suggest an association between CLZ treatment and comorbid OCS. However, a possible confounding effect due to the selection of specific SGAs for specific subgroups of patients has to be considered.

Noteworthy, in some cases an alleviation of OCS severity after the addition of CLZ (Peters and de, 2009), an increase in CLZ dosage (Lykouras et al., 2003) or start with OLZ treatment (van Nimwegen et al., 2008; Poyurovsky, 2013) has been observed. Regarding these contradicting findings, some important aspects should be discussed. One explanation relates to the above mentioned diagnostic difficulties to differentiation between OCS and delusional or catatonic symptoms (Table 1). Patients with schizophrenia, who show obsessive ruminations or stereotypic thoughts during acute psychosis or repetitive ritualized behavior clearly related to the patient's primary psychotic condition might indeed benefit from treatment with CLZ. Thus, careful diagnostic evaluations are necessary. Furthermore, anti-obsessive effects of antipsychotics have also been reported in primary OCD, including OLZ, especially in cases with treatment-resistance to serotonergic antidepressants (Bloch et al., 2006; Bandelow et al., 2008; Dold et al., 2011; Muscatello et al., 2011). Nevertheless, even in treatment-resistant OCD current treatment guidelines do not recommend CLZ as an augmentation strategy.

#### Associations between the duration of treatment and OCS severity

Correlations between pharmacological variables and OCS provide further support for proposed causal interactions. Lin et al. (2006) compared CLZ-treated patients with and without comorbid OCS and found significantly longer CLZ treatment periods for the comorbid group, but no difference in duration of illness. Schirmbeck et al. reported a positive correlation between OCS severity and duration of CLZ-treatment (Schirmbeck et al., 2011). Accordingly, de Haan et al. reported this association for OLZ (de Haan et al., 2002).

### Association between dosage and blood serum levels and OCS severity

Several authors demonstrated positive correlations between dose or serum levels of CLZ and severity of OCS (Lin et al., 2006; Reznik et al., 2004; Mukhopadhaya et al., 2009; Schirmbeck et al., 2011). Similarly, a reduction of daily CLZ-dosage, for instance through the combinations with another SGA, such as APZ, resulted in an alleviation of OCS severity (Rocha and Hara, 2006; Zink et al., 2006; Englisch et al., 2009). This observed effect might represent both a reduction of the suggested dose-related side effect of CLZ and/or a consequence of inherent anti-obsessive effects of APZ due to its partial dopaminergic and serotonergic agonism. The latter assumption was supported by a *placebo* controlled randomized trial, which showed reduced OCS severity after combination with APZ, but unchanged CLZ dose during the course of the study (Chang et al., 2008).

#### Differential effects of SGAs on the course of OCS

A recent longitudinal study revealed differential effects of antipsychotic agents on comorbid OCS. Within a 12 months observational period, changes in YBOCS scores significantly differed between two pharmacologically diverse groups (completer analysis: *p* = 0.006; full sample analysis: *p* = 0.007). Whereas the CLZ/OLZ group showed persistently high OCS severity over time, the AMS/APZ group reported further decrease of the initially low YBOCS-scores (Schirmbeck et al., 2013).

In conclusion, reported data show strong associations between comorbid OCS in schizophrenia and mainly antiserotonergic SGAs, most importantly CLZ. The published epidemiological and pharmacological evidence hint toward causal interactions, suggesting CLZ's strong inherent antiserotonergic properties (Steingard et al., 1993; Joober and Boksa, 2010; Kang and Simpson, 2010), most importantly the antagonism at 5-HT1C, 5-HT2A and 5HT2C receptors (Coward, 1992; Meltzer, 1994; Meltzer and Huang, 2008) as an relevant underlying mechanism. Low affinities to dopamine receptors result in a very small ratio of dopaminergic/serotonergic receptor blockade, which largely differs from SGAs such as AMS or APZ (Scatton et al., 1997; Shapiro et al., 2003; Correll, 2008). In addition, reciprocal interactions of dopaminergic and serotonergic neurotransmission with glutamatergic and GABAergic functions might play an important role (Lopez-Gil et al., 2010). Thus, pharmacotherapy constitutes a relevant environmental factor, which might exert pro-obsessive effects in schizophrenia patients. Within a broader perspective, additional questions arise concerning predisposing factors. These might comprise patient-inherent characteristics (neurocognitive profile, the subtype of psychosis, the stage of the illness, any kind of affective comorbidity or a family history for anxiety disorders) and the individual genetic disposition.

### Genetic disposition

Associations with the gene *SLC1A1* have consistently been replicated in primary OCD patients. Based on these findings a South Korean research group investigated the genetic risk to develop second-onset OCS during treatment with SGAs (Kwon et al., 2009b). Kwon et al evaluated associations between specific SNPs of the candidate gene *SLC1A1* and SGA-induced OCS and showed strong associations with the A/C/G dominant haplotype rs2228622 / rs3780413 / rs37801412. The odds ratio of 3.96 indicated an almost 4 times higher likelihood for patients, who carried this A/C/G haplotype to suffer from SGA-induced OCS. Neither the gene *SLC1A1* nor its chromosomal region has been associated with vulnerability to schizophrenia spectrum disorders (Deng et al., 2007). The same group further described a genetic interaction of the *SLC1A1* polymorphism with variants in the gene *DLGAP3* (disks large associated protein 3) and a link to SGA-induced OCS (Ryu et al., 2011). In addition, Cai et al reported on an interaction of SNPs in SLC1A1 and the type 2B subunit of the N-methyl-D-aspartate receptor gene (GRIN2B), as well as interactions with the YBOCS score in Chinese patients (Cai et al., 2013).

A replication approach of the results obtained by Kwon et al. (2009b) was conducted in 103 schizophrenia patients of European descent treated with SGAs. However, the described finding could not be reproduced, neither in single marker, nor in haplotype analyses. Because no genetic associations between *SLC1A1* polymorphisms and OCS were found within the power limits of this study, much larger samples seem necessary to untangle the interplay of pharmacological and genetic risk factors for OCS in schizophrenia (Schirmbeck et al., 2012a). The brain derived neurotrophic factor (*BDNF*) was recently proposed as a third candidate gene, because the Val66Met polymorphism was found to be associated with OCS in schizophrenia (Hashim et al., 2012). So far, independent replication approaches regarding *BDNF*, *DLGAP3* and GRIN2B have not been conducted.

## Research perspectives

### GxEI on a second level of complexity

GxEIs are core elements within current theories of schizophrenia (van Os and Kapur, 2009; van Os et al., 2008, 2010), depression (Keers and Uher, 2012), anxiety disorders (Gregory et al., 2008; Nugent et al., 2011) and OCD (Nicolini et al., 2009; Pauls, 2010). High rates of bi-directional comorbidities lead to the obvious question, if these co-occurrences could also be explained by common GxEIs. One example of this experimental psychopathology has been illustrated by the described investigation of the risk to develop secondary OCS during treatment with SGAs. Here, the environmental factor is represented in the pharmacological treatment of schizophrenia with pro-obsessive SGAs.

As stated in chapter 4, SGAs increase the risk for secondary OCS via a pharmacodynamic mechanism. Independently, a set of SNPs within the gene *SLC1A1* seem to predispose to OCD. However, the initially reported high odds ratio by Kwon et al. (2009b) could not be replicated in a similar study performed with European patients (Schirmbeck et al., 2012a). Thus, the general genetic background of a patient (Asian or European) might be of importance when a specific SGA (balance between dopaminergic and serotonerigic blockade) is introduced as the treatment of choice. Furthermore, gene-x-gene interactions (SNPs in *SLC1A1*, *BDNF*, DLGAP3, and GRIN2B) have been suggested as further influencing factors (Ryu et al., 2011; Hashim et al., 2012) and should be considered in forthcoming studies. It is an important progress in recent neurobiological research to investigate how the interaction of these factors might influence the propensity of schizophrenia patients to suffer from comorbid OCS when being treated with SGAs.

Future progress might depend on two aspects: First, well defined homogeneous clinical cohorts should be defined to reduce the number of possible confounding causal factors to a minimum. Considering the order of symptom onset, the clinical course and the applied treatment for sample characterization might be helpful. Second, much larger cohorts have to be recruited in multicenter studies to investigate possible genetic risk constellations. If power analyses would be based on the much smaller genetic-risk estimations for the gene *SLC1A1* in the European sample (Schirmbeck et al., 2012a), group size calculations result in about five thousand participants, which would be necessary for replication.

Besides pharmacological treatment as a relevant factor, further non-pharmacological environmental factors could play an important role in the development of OCS in schizophrenia. Such factors might include, psychosocial stress induced by critical life events, interpersonal factors, changes of the vocational situation or the present state of general physical health. In addition, the reciprocal interaction and possible causal directions between OCS and psychotic positive, negative and cognitive symptoms of schizophrenia must be unraveled and considered. One important tool to unravel the interdependence of these variables are the above described experience sampling approaches. These allow to investigate the individual symptom variability in real life situations on a day to day basis.

Collected data will help to identify the time course of symptom-changes and its relation to important environmental factors. These studies are currently planned and will hopefully result in an improved understanding of etiological factors influencing the course of OCS in schizophrenia. Within this context it will also be desirable to collect DNA samples in order to analyse possible predisposing effects of the above mentioned polymorphisms to experience the development or aggravation of OCS after being exposed to stressful life events. Thus, combining experience sampling and genetic characterizations might markedly improve our insight into GxEI.

### Therapy

At present, pharmacological treatment interventions, most importantly combination as well as augmentation strategies, have been suggested to improve OCS in the highly impaired comorbid group (Schirmbeck et al., 2013). To address possible pro-obsessive effects of predominantly anti-serotonergic SGAs, the add-on of mainly dopaminergic SGAs such as AMS and APZ has been proposed (Connor et al., 2005; Zink et al., 2006; Englisch and Zink, 2008; Kim et al., 2008; Yang et al., 2008; Englisch et al., 2009; Muscatello et al., 2011). In addition, the augmentation with serotonergic antidepressants has been evaluated, for example with the tricyclic antidepressant clomipramine (Berman et al., 1995b) or with SSRIs, most often fluvoxamine (Poyurovsky et al., 1999b; Reznik and Sirota, 2000; Hwang et al., 2009). Results of these trials have been inconsistent with some studies failing to observe the intended effects of OCS reduction. Furthermore, additive anticholinergic side effects and pharmacokinetic interactions have to be considered. Finally, first promising results were published reporting on the augmentation with mood stabilizers such as valproic acid (Zink et al., 2007; Canas et al., 2012) or lamotrigine (Poyurovsky et al., 2010; Rodriguez et al., 2010).

So far, very limited data exists on the efficacy and safety of cognitive behavioral therapy (CBT) for this group of patients. The small number of case reports and case series can hardly be reconciled with the fact, that CBT including exposure and response prevention is considered treatment of first choice for primary OCD with remarkably high effect sizes (Gava et al., 2007; Koran et al., 2007; Rosa-Alcazar et al., 2008; Kuelz and Voderholzer, 2011). With one exception, currently available CBT manuals for OCD do not provide guidelines for the treatment of OCS in schizophrenia (Emmelkamp and van Oppen, 2000; Lakatos and Reinecker, 2007; Oelkers et al., 2007; Foerstner et al., 2011). However, a summary of the published reports on 30 comorbid patients (Schirmbeck et al., 2013), who were treated with CBT including exposure elements or just exposure and response prevention alone showed favorable outcome measures with significant reduction of OCD severity in 24 patients. In the included case series by Tundo et al. (2012) 52% of investigated individuals were classified as “much or very much” improved, 33% as responders and 19% as remitters. The available evidence of CBT for OCS in schizophrenia is certainly limited by the small case numbers and further controlled clinical trials are needed. However, despite adverse clinical outcomes in 10%, and a total dropout rate of 20%, preliminary results suggest meaningful and marked reduction of OCS severity in 80% of participants (Schirmbeck et al., 2013). Table 3 summarizes possible pharmacological and non-pharmacological approaches and current evidence of empirical support.

**Table 3:** **Therapeutic approaches**.

Early recognition and monitoring	Definition of at-risk-constellations.
	Monitoring of subclinical levels of OCS or beginning cognitive impairment using sensitive sets of neurocognitive tests (Schirmbeck et al., 2011, 2012b).
Polypharmacy	Augmentation with antidepressants: Clomipramine, fluvoxamine and other SSRIs. [Level of evidence: RCTs, CS, CR] (Berman et al., 1995b; Poyurovsky et al., 1999b; Reznik and Sirota, 2000).
	Caveat: Additive (anticholinergic) side effects and pharmacokinetic interactions
	Augmentation with mood stabilizers (lamotrigine, valproic acid) aiming at a reduction of SGA-dosage to minimally sufficient levels [Level of evidence: CS, CR] (Zink et al., 2007; Poyurovsky et al., 2010; Rodriguez et al., 2010; Canas et al., 2012).
	Combination of pro-obsessive SGAs with neutral or anti-obsessive SGAs (amisulpride, aripiprazole) in order to reduce the clozapine-dosage to minimally sufficient levels [Level of evidence: RCT, CS, CR].
	(Connor et al., 2005; Zink et al., 2006; Englisch and Zink, 2008; Kim et al., 2008; Yang et al., 2008; Englisch et al., 2009; Muscatello et al., 2011)
Psychotherapy	Cognitive behavioral therapy involving exposure and response prevention [Level of evidence: CS, CR] (Schirmbeck and Zink, 2012; Tundo et al., 2012).

*Summary of therapeutic approaches for schizophrenia patients with comorbid OCS or OCD. The current level of empirical evidence is indicated in square brackets*.

Abbreviations *CR* *case report* *CS* *case series* *RCT* *randomized controlled trial*.

## Conclusions

The summarized data substantiate the conclusions that OCS is a very frequent and relevant comorbid burden in schizophrenia. The clinical presentation of the co-occurrence is very diverse, suggesting different subgroups with heterogeneous pathogenic mechanisms. First insight into GxEI has been achieved for the subgroup of patients who experienced second-onset OCS during treatment with SGAs. In the future, a broader set of environmental variables, including non-pharmacological factors, and further genetic risk-constellations should be analysed, starting in the ARMS. In perspective, this will not only improve the risk prediction regarding comorbid OCS, but also early recognition and monitoring of emerging symptoms. Research within this field will further provide the individual framework of predisposing and disease-provoking factors with immediate impact for pharmacological and CBT approaches.

### Conflict of interest statement

Frederike Schirmbeck was supported by an unrestricted scientific grant of Evangelisches Studienwerk. Mathias Zink received unrestricted scientific grants of the European Research Advisory Board (ERAB), German Research Foundation (DFG), Pfizer Pharma GmbH, Servier and Bristol Myers Squibb Pharmaceuticals; further speaker and travel grants were provided from Astra Zeneca, Lilly, Pfizer Pharma GmbH, Bristol Myers Squibb Pharmaceuticals, Servier, Otsuka, Roche and Janssen Cilag.

## Abbreviations

AMS: amisulpride
APZ: aripiprazole
ARMS: at risk mental state
BDNF: brain derived neurotrophic factor
CBT: cognitive behavioral therapy
CLZ: clozapine
DLGAP3: disks large associated protein 3
GxEI: Gene and environment interaction
OCS: obsessive-compulsive symptoms
OCD: obsessive-compulsive disorder
OLZ: olanzapine
SGA: second generation antipsychotics
SLC1A1: solute carrier family gene 1A1
SNP: single nucleotide polymorphism
SSRI: selective serotonin reuptake inhibitor.
